# The Behavioral and Neurochemical Aspects of the Interaction between Antidepressants and Unpredictable Chronic Mild Stress

**DOI:** 10.32607/actanaturae.10942

**Published:** 2020

**Authors:** N. V. Kudryashov, T. S. Kalinina, A. A. Shimshirt, A. V. Volkova, V. B. Narkevich, P. L. Naplekova, K. A. Kasabov, V. S. Kudrin, T. A. Voronina, V. P. Fisenko

**Affiliations:** Federal State Budgetary Institution «Research Zakusov Institute of Pharmacology», Moscow, 125315 Russia; Sechenov First Moscow State Medical University (Sechenov University), Moscow, 119991 Russia; N.K. Koltsov Institute of Developmental Biology RAS, Moscow, 119334 Russia

**Keywords:** chronic mild stress, amitriptyline, fluoxetine, monoamines, forced swim, mice

## Abstract

The behavioral and neurochemical effects of amitriptyline (10 mg/kg, i.p.) and fluoxetine (20 mg/kg, i.p.) after single and chronic administration in the setting of unpredictable mild stress in outbred ICR (CD-1) mice were studied. After a 28-day exposure to stress, we observed an increase in depressive reaction in a forced swim test in mice, as well as reduced hippocampal levels of serotonin (5-hydroxytryptamine, 5-HT) and 5-hydroxyindoleacetic acid (5-HIAA) and an increased hypothalamic level of noradrenaline (NA). Single and chronic administration of amitriptyline and fluoxetine shortened the immobility period and increased the time corresponding to active swimming in the forced swim test. The antidepressant-like effect of fluoxetine – but not of amitriptyline – after a single injection coincided with an increase in the 5-HT turnover in the hippocampus. Chronic administration of the antidepressants increased the hypothalamic levels of NA. Thus, the antidepressant- like effect of amitriptyline and fluoxetine may result from an enhancement of the stress-dependent adaptive mechanisms depleted by chronic stress.

## INTRODUCTION

The main goals pursued in the pharmacotherapy of major depressive disorders (MDD) using antidepressants is to achieve remission and maintain it over time [1]. However, several clinical trials have shown that only one-third of patients undergoing drug therapy using antidepressants achieve stable remission after a single course of treatment [1, 2]. In line with this observation, the concept of treatment-resistant depression was proposed in order to describe a depressive disorder that could not be put into stable remission after pharmacotherapy [1].

Stress has been singled out as being among the other factors causing the development of treatment-resistant depression. Young et al. [3] have reported that non-response to treatment with fluoxetine is associated with the hyperactivity of the hypothalamic–pituitary– adrenal (HPA) axis. Moreover, MDD patients with Cushing’s disease, accompanied by the overproduction of adrenal hormones, also respond poorly to treatment with classical MDD pharmacotherapy [1]. Finally, the polymorphism in the promoter region of the gene encoding the serotonin transporter is linked to the vulnerability to stressful life events that results in depression and resistance to antidepressants [4].

Chronic mild stress (CMS) is an animal model of depression which was developed more than 20 years ago [5]. Different modifications of CMS are routinely used to mimic the connection between stressful events and depression [5-9]. In humans, long-term exposure to uncontrollable and unpredictable life stressors is often regarded as an underlying cause of depressive disorders [8]. In a model of CMS, rodents are unpredictably exposed to mild stressors for several (2–7) weeks. These stress factors can be either social or physiological (immobilization, isolation, food or water deprivation, circadian rhythm abnormalities, dirty home cages, damp sawdust, sounds of predators, etc.) [5]. In CMS-treated rodents, motivational deficiency in sucrose solution intake is regarded as anhedonia, while the longer immobility time in the forced swim test (FST) is seen as an analog of dysphoria. Sexual dysfunction, anxiety, weight loss, and decreased exploratory activity are often used as depression-like signs in rodents exposed to a CMS procedure [7, 9, 10].

Numerous studies have used a CMS protocol to assess the antidepressant-like effects of drugs; however, drug administration usually starts after a long-term exposure to CMS [11-14]. In the present study, we focused on the interaction between stressful events and the effects of antidepressants. Therefore, chronic administration of amitriptyline and fluoxetine was started simultaneously with the CMS protocol. This experimental design may prove useful in assessing the behavioral and neurochemical effects of amitriptyline and fluoxetine in the dynamics of development of a response to CMS. It may also provide an answer to the question of possible structure-specific neurochemical interactions between chronic stress and antidepressants.

## EXPERIMENTAL

**Animals**

The experiments were conducted using 60 male ICR (CD-1) mice weighing 25–35 g (Research Center of Biomedical Technology, Federal Medical and Biological Agency, Russia). The animals were group-housed under standard conditions, with a 12-h dark–light cycle at a temperature of 22 ± 2°C and ad libitum access to granulated chow (MEST, Russia) and water. All animal treatments and experimental procedures were carried out in accordance with the Good Laboratory Practice approved by the Ministry of Public Health of Russia (Supplement to order N 199n of April 1, 2016).

**Drugs**

Fluoxetine hydrochloride (Sigma Aldrich) was dissolved in 0.9% saline containing Tween-80 (Sigma Aldrich). Amitriptyline (Moscow Endocrine Plant, Russia) was dissolved in 0.9% saline. The fluoxetine (20.0 mg/kg) and amitriptyline (10.0 mg/kg) solutions were injected intraperitoneally (i.p.). The antidepressants doses were selected based on earlier studies [15]. The following factors were taken into account when selecting the drugs: (1) amitriptyline is a tricyclic antidepressant, a non-selective inhibitor of monoamine reuptake characterized by the predominance of sedation; fluoxetine is a selective serotonin reuptake inhibitor exhibiting a strong stimulant effect; (2) amitriptyline and fluoxetine are reference drugs that are conventionally used in preclinical studies; (3) fluoxetine may enhance neurosteroidogenesis in the CNS, which is part of the adaptive response to stress. This is an additional reason for assessing the interaction between fluoxetine and chronic stress [16].

**Unpredictable chronic mild stress.**

The animals were exposed to chronic stressors in a quasi- random manner (wet bedding, dirty boxes, water deprivation, reduction in daylight hours, etc.) within four weeks (*Table 1*).

**Table 1:** The UCMS protocol

day 1	day 2	day 3	day 4	day 5	day 6	day 7
Wet bedding 11:00 – 15:00	Lack of light 16:00 → 11:00		Cat odor 13:00 – 14:00	Overcrowded home cages 9:00 – 17:00	White noise 12:00 – 15:00	Dirty home cages
day 8	day 9	day 11	day 12	day 13	day 14	day 15
Food deprivation 12:00 → 12:00		Tilting of himecages 16:00 – 20:00	Cat odor 11:30 – 15:30	Food deprivation 12:00 → 12:00		Cat odor 10:00 – 11:00
day 15	day 16	day 17	day 18	day 19	day 20	day 21
Wet bedding 9:00 – 13:00	White noise 13:00 – 16:00	Empty water bottles 11:00 – 15:00	Cat odor 9:00 – 10:00	Cat odor 12:30 – 13:30	Overcrowded home cages 9:00 – 13:00	Dirty home cages
day 22	day 23	day 24	day 25	day 26	day 27	day 28
White noise 10:00 – 15:00	Water deprivation 12:00 → 9:00		Cat odor 11:00 – 12:00	Overcrowded home cages 15:00 – 6:00	Acute forced swim (5 min)	The forced swim test and neurochemical measurements
		Water deprivation 12:00 → 9:00				

**Forced swim test**

The antidepressant activity was studied in a modified version of the forced swim test in mice [16]. Cylindrical transparent Plexiglas tanks (30 cm height × 10 cm diameter) were filled with water (25 ± 1°C) up to 20 cm from the bottom. Twenty-four hours before the test, the animals were placed in the tanks filled with water for 5 min. Before being returned to their home cages, the animals were gently dried with paper towels. On test day, the mice were put in the cylinders for a 5-min swim session, which was video-recorded with recording of the duration of the climb, active swimming, and immobility periods. Climbing was defined as the upward movement of forelimbs along the tank walls. Swimming was defined as active use of forelimbs (while not lifting them above the water level) to move forward, towards the tank center or walls. Immobility was defined as lack of activity other than that required from the animal to keep its head above water: tail movements and limited limb movements.

**Neurochemical measurements**

Decapitation was performed 30 min after the behavior test. The brain structures (the medial prefrontal cortex (mPFC), hypothalamus and hippocampus) were dissected on an ice-cold surface (+4°C), weighed, and immediately stored in liquid nitrogen. Tissue samples were homogenized in 0.1 N perchloric acid with 0.25 nmol/ml 3,4-dihydroxybenzoic acid (1 : 20) as an internal standard and centrifuged (10 000 *g *× 10 min, 4°C). The supernatant was analyzed by high-performance liquid chromatography, coupled with electrochemical detection (HPLC/ECD). Monoamines and their metabolites were detected using a glassy carbon electrode set at +0.85 V against the Ag/AgCl reference electrode using an LC-4B electrochemical detector (Bioanalytical Systems, USA). The mobile phase contained a 0.1 M citrate-phosphate buffer, 1.1 mM 1-octanesulfonic acid, 0.1 mM ethylenediaminetetraacetate (EDTA), and 9% acetonitrile; pH was adjusted to 3.0 with 6 M KOH. The studied substances were separated on a Reprosil C18 analytical reversed-phase column (pore size, 4 μm; 100 × 4 mm) (Dr. Maisch GMBH, Germany) at a flow rate of 1.0 ml/min. All the reagents used in the mobile phase were of analytical grade. The monoamine levels in the experimental sample were quantified by external standard curve calibration using the peak area for quantification. Sample analysis was performed using the MULTICHROM 1.5 software (Ampersand, Russia) [17].

**Statistical analysis**

Analysis was performed using the GraphPad Prizm7.0 software (GraphPad Software Inc., USA). The normal distribution of the data was evaluated using the Shapiro– Wilk test. The results are presented as means ± SEM. The significance of intergroup differences was estimated by one-way analysis of the variance (ANOVA), followed by a post-hoc Newman–Keuls test.

## RESULTS

**Behavioral changes**

**Fig. 1:**
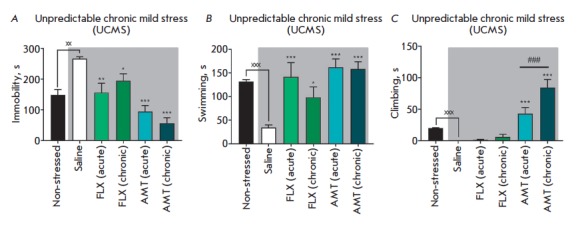
Effects of amitriptyline (10 mg/kg) and fluoxetine (20 mg/kg) under UCMS in the forced swim test in mice.* A *– duration of immobility period; *B *– duration of swimming; *C *– duration of climbing. The data are presented as M ± SEM; Without stress and control – 0.9% NaCl (0.1 ml/10 g body weight); UMCS – unpredictable chronic mild stress; FLX – fluoxetine, AMT – amitriptyline; acute – single administration; chr. – chronic administration during 28 days. ### – *p* < 0.001 compared with the non-stressed group; * – *p* < 0.05; ** *p* < 0.01; *** – *p* < 0.001 compared with the saline group under stress; xxx – *p* < 0.001; xx – *p* < 0.01 compared with single administration

Mice exposed to UCMS exhibited some behavior changes compared to the control animals: suppression of climbing activity (*p* < 0.001), a 3.8-fold decrease in swimming duration (*p* < 0.001), and a 1.8-fold increase in the duration of the immobility period (*p* < 0.001) (*Fig. 1*).

Single injection of amitriptyline after UCMS, as well as a 28-day treatment regiment amidst UCMS, restored climbing activity (*p* < 0.001), increased the swimming time 4.6- to 4.7-fold (*p * < 0.01), and reduced the duration of immobility 2.8- to 4.7-fold (*p* < 0.001). Chronic treatment with amitriptyline enhanced climbing activity compared to single injection of the drug (*p* < 0.001).

**Fig. 2:**
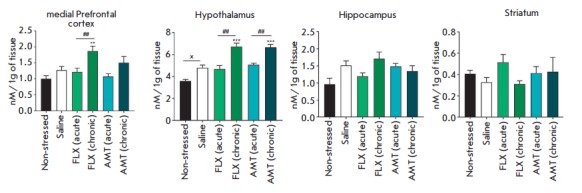
The influence of amitriptyline (10 mg/kg) and fluoxetine (20 mg/kg) on NA level in the brain of mice exposed to UCMS. x – *p* < 0.05 compared with the non-stressed group. **; *** – *p* < 0.01; *p* < 0.001 compared with UCMS, respectively. ## – *p* < 0.01 compared with acute administration

Single injection of fluoxetine under UCMS was accompanied by a 4.1-fold increase in swimming time (*p* < 0.001) and reduction of immobility time 1.7-fold (*p* < 0.01). Chronic treatment with amitriptyline increased swimming duration 2.8-fold (*p* < 0.05) and reduced the immobility time 1.4-fold (*p * < 0.05).

**Fig. 3:**
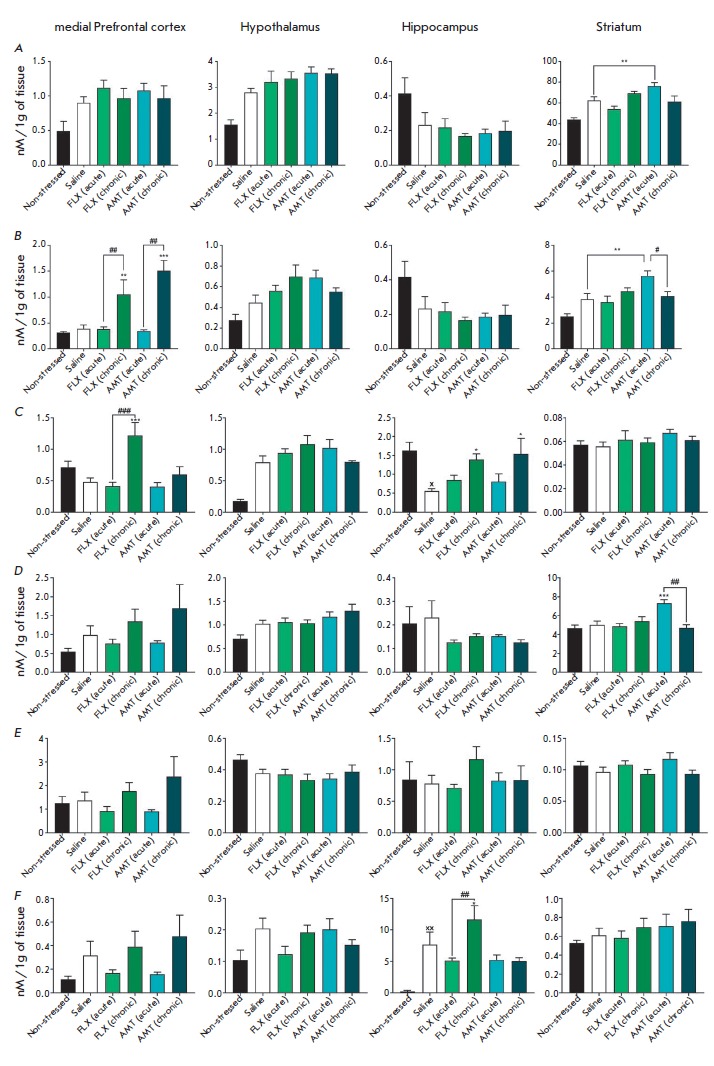
The influence of amitriptyline (10 mg/kg) and fluoxetine (20 mg/kg) on the DA level (*A*), the DOPAC level (*B*), the DOPAC/ DA ratio (*C*), the HVA level (*D*), the HVA/ DA ratio (*E*), and the 3-MT level (*F*) in the brains of mice exposed to UCMS. x; xx – *p* < 0.05;* p* < 0.01 compared with the non-stressed group. *; **; *** – *p* < 0.05;* p* < 0.01;* p* < 0.001 compared with the UCMS. #; ##; ### – *p* < 0.05; *p* < 0.01; *p* < 0.001 compared with single administration

**Fig. 4:**
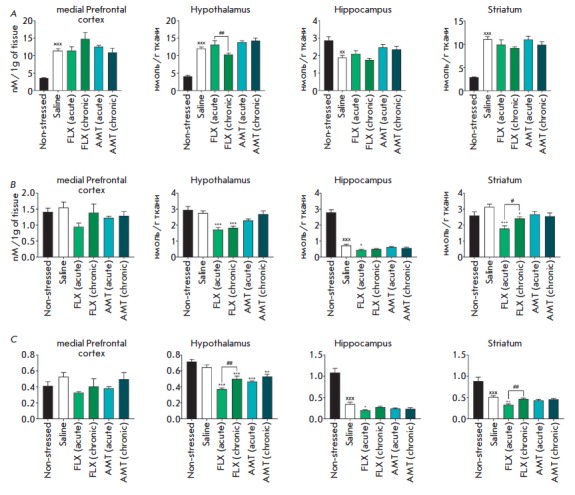
The influence of amitriptyline (10 mg/kg) and fluoxetine (20 mg/kg) on the 5-HT level (*A*), the 5-HIAA level (*B*), the 5-HIAA/5-HT ratio (*C*) in the brains of mice exposed to UCMS. xx; xxx – *p* < 0.01; *p* < 0.001 compared with the non-stressed group. *; **; *** – *p* < 0.05; *p* < 0.01; *p* < 0.001 compared with UCMS. #; ## – *p* < 0.05; *p* < 0.01 compared with single administration

*Neurochemical changes*. The dynamics of neurochemical changes in the levels of neuroamines and their metabolites in the mPFC, hypothalamus, and hippocampus are presented in *Fig. 2*, *Fig. 3*, *Fig. 4*. The most significant alterations were as follows: a decrease in the 5-HT level (by 30%, *p* < 0.01) and its turnover (by 67%, *p* < 0.001) in the hippocampus; an increase in the hypothalamic levels of NA (by 33.7%, *p * < 0.05) and a reduction of the DOPAC/DA ratio in the hippocampus (by 49.5%, *p* < 0.05) in mice subjected to UCMS compared to those in the control group.

Single and chronic administration of the studied antidepressants under UCMS tended to increase the 5-HT levels and reduce the 5-HT turnover in the hippocampus, compared to the UCMS group. Chronic, but not single, administration of amitriptyline and fluoxetine increased the hypothalamic levels of NA (by 39.5 and 39.6%, respectively; *p* < 0.001) and the DOPAC/ DA ratio in the hippocampus (by 150 and 133%, respectively; *p* < 0.05), compared to those in the UCMS group. Chronic treatment with fluoxetine also increased the DOPAC/DA ratio in the mPFC (by 140%, *p* < 0.001).

## DISCUSSION

Exposure to UCMS enhanced depressive-like behavior, which is consistent with data in the literature and was also observed after subchronic corticosterone administration [7, 9, 18]. Neurochemical changes caused by UCMS were detected in all the aforementioned brain structures, although each had distinctive characteristics. Thus, only an elevated 5-HT level was observed in the mPFC, while the hypothalamic levels of both NA and 5-HT were increased. In the striatum, the 5-HT level was elevated, while its turnover declined. In the hippocampus of mice exposed to UCMS, the level and turnover of 5-HT was reduced and DA metabolism, determined by DOPAC turnover, was decreased. Meanwhile, the level of another metabolite, 3-MT (which indicates the decline in the dopamine transporter function), was elevated.

Hence, the most significant changes in the monoamine levels and turnover in mice with depressive-like reactions were detected in the hippocampus. One of the most notable observations was related to the fact that the 5-HT levels were increased in all studied brain structures except for the hippocampus, where the 5-HT levels were reduced, as has been the case under chronic noise [19] and immobilization stress [20]. Several precursor studies have reported that the 5-HT levels decrease after UCMS, instead of increase [21-24] (including experiments with the forced swim test as an element of chronic stress exposure) [22-24]. This contradiction can be explained by the fact that mice with genetic deficiencies were used and that the forced swim stress had not been the final procedure before the monoamine levels were evaluated in those studies, while in our study mice were decapitated 30 min after the forced swim procedure. It has been recognized, however, that single and repeated exposure to forced swimming increases the intracellular levels of 5-HT and 5-HIAA in different brain structures in rodents [25-28], whereas varied acute stressors (electric shock, tail pinch, etc.) enhance the activity of 5-HT neurons in the midbrain nuclei and increase 5-HT levels in the amygdala, mPFC, raphe nuclei, and hippocampus [29]. In our study, presumably, the hippocampal response to forced swim stress, which was the last component in a battery of stressors, coincided with the reaction to chronic stress and was characterized by a depletion of 5-HT. Moreover, these results are consistent with the published data on an increased MAO-A activity and depletion of 5-HT after a CMS procedure and decrease in 5-HIAA in the cerebrospinal fluid of a depressed patient [29].

These neurochemical alterations in the serotonergic system may be associated with the impact of chronic stress on adult hippocampal neurogenesis. De Andrade et al. [30] explored adult neurogenesis and stress interactions and reported that a CMS procedure (for 14 consecutive days) reduced the number of doublecortin- positive cells in the dorsal and ventral hippocampuses and increased the serum levels of corticosterone in rats. Furthermore, these neurogenic alterations correlate with a display of anxiety-like behavior. Mineur et al. [31] also observed a reduced survival rate of newborn neurons in both the hippocampus and the subventricular zone in mice exposed to the CMS procedure.

On the other hand, 5-HT plays an important role in the maintaining of homeostasis, while the decline in the 5-HT level correlates with symptomatic anxiety and depression disorders. To our knowledge, 5-HT significantly contributes to adult hippocampal neurogenesis [32]. There is evidence of a modulatory role for 5-HT in adult hippocampal neurogenesis, based on pharmacological manipulations with serotonergic neurons in the raphe nuclei or inhibition of 5-HT synthesis in the CNS [33, 34]. Inhibition of 5-HT synthesis leads to a steep decline in the proliferation and survival rate of adult hippocampal progenitors and reduces the number of doublecortin-positive cells in the neurogenic niche in the hippocampus [34]. The destruction of 5-HT fibers in the raphe nuclei was accompanied by a decrease in the number of newly generated granule cells labeled with bromodeoxyuridine (BrdU) in the dentate gyrus [33]. Finally, a wide range of 5-HT receptors have a direct or indirect impact on different phases of adult neurogenesis in the dentate gyrus [32].

We suspect that there exists a correlation between the selective decrease in the hippocampal level of 5-HT and the depressive-like behavior of mice in the forced swim test. Therefore, fluctuations in the hippocampal level of 5-HT may be a neurochemical marker of the intensity of the proliferative phase of neurogenesis under stress of varying duration.

The results of our study revealed increased hypothalamic levels of NA after the exposure to UCMS. The increased levels of NA and its metabolites were observed after exposure to UCMS for 14 days [21, 35], but not for 54–60 days [36, 37]. We suppose that the increase in the hypothalamic levels of NA can be regarded as a response to chronic stress; these changes are indicative of an adaptive activation of the mechanisms that maintain neurogenesis. This increase in the NA levels after a short-term CMS procedure (2–4 weeks) coincides with another process; namely, stem cell proliferation in an adult hippocampus [38-40]. Direct activation of β3-adrenoreceptors is known to increase the number of proliferating cells in an adult hippocampus [41]. Moreover, this is in line with the regulatory role we suspect is played by the hypothalamus in adult neurogenesis [42, 43]. Taken together, these facts may be indicative of an interconnection between the peak of NA levels in the hypothalamus and activation of the adaptive repair mechanisms of the CNS, which may decline after longterm (over 4 weeks) exposure to stress.

It draws one’s attention that in all the analyzed brain structures, exposure to UCMS did not significantly affect the levels of DA and its metabolites, except for the manifold increase in the 3-MT level and selective decrease in the DOPAC/DA turnover in the hippocampus.

Amitriptyline and fluoxetine exhibited their antidepressant properties after single, as well as chronic, administration regardless of exposure to stress, but these manifestations were different. Hence, restoration of climbing activity was observed after single injection of amitriptyline, but not fluoxetine. This effect was enhanced after chronic administration of the antidepressant.

Certain characteristic features were noted among the neurochemical effects of the antidepressants: (1) the antagonistic effects with respect to UCMS, (2) the enhancement of the changes caused by UCMS, (3) the influence on the parameters which had not been affected by the stress procedure, and (4) the effects of chronic (but not single) administration. As previously mentioned, UCMS decreases the DOPAC/DA ratio in the hippocampus of stressed mice compared to that in stress-free animals. Chronic, but not single, administration of amitriptyline, as well as fluoxetine restored this parameter to the reference values. Moreover, chronic administration of both drugs caused an even more marked increase in the hypothalamic levels of NA than after exposure to UCMS. Chronic treatment with fluoxetine also increased NA levels in the mPFC, which corresponds to the conclusions drawn by R. Xue et al. [44]

However, notwithstanding the ubiquitous changes in the serotonin content in all the studied brain structures after UCMS, neither single nor chronic administration of the antidepressants influenced this parameter. Meanwhile, fluoxetine, but not amitriptyline, reduced the levels of serotonin metabolites in the hypothalamus and the striatum in non-stressed mice (which were not affected by UCMS) and enhanced the effect of UCMS on the serotonin turnover in the hippocampus and the striatum, which is consistent with the ability of the antidepressant to selectively influence the serotonin transporter. After single administration, fluoxetine reversed the effects on the 3-MT level caused by UCMS.

Thus, the behavioral effects caused by single or chronic administration of the antidepressants in mice exposed to UCMS are associated with changes in the levels and turnover of monoamines. However, fluoxetine and amitriptyline share one feature: when administered chronically, they can restore the DOPAC/DA ratio to its control values and potentiate the increase in the hypothalamic NA level, which provides ground for one to view these neurochemical changes as a substrate for the development of the antidepressant effect of the studied drugs in a forced swim test.

It has been reported that antidepressants increase the number of mitoses in the subgranular zone of the dentate gyrus in the hippocampal formation 2–4 times [32, 45]. Acute stress causes similar changes, while exposure to chronic stress reduces the mitotic number of progenitor cells [30, 31].

The CMS procedure for 28 consecutive days significantly reduced the DOPAC/DA ratio in the hippocampus, while in the mPFC such changes were just a trend. Chronic, but not acute, treatment with fluoxetine increased the DOPAC/DA ratio in the hippocampus and tended to increase the same ratio in these structures after chronic administration of amitriptyline. These neurochemical alterations may be a sign that there is a correlation between the impacts of CMS, fluoxetine, and amitriptyline on adult hippocampal neurogenesis.

Taken together, our observations and analysis of the published data provide evidence for a possible role played by the DOPAC/DA ratio as a marker of neurogenesis in an adult mammalian brain, which should be considered in the context of future experimental studies. Thus, an increase in this ratio may be evidence of positive regulation of precursor cell proliferation, while a decrease in the DOPAC/DA ratio may, inversely, be indicative of neurogenesis suppression. The exposure to several different factors (e.g., 1-bromopropane inhalation or intraperitoneal administration of the pro-neurotoxin 1-methyl-4-phenyl-1,2,3,6-tetrahydropyridine (MPTP)) simultaneously causes neurogenesis suppression in an adult mammalian brain and alters the DOPAC/DA turnover [47-49].

Intoxication with 1-bromopropane is associated with the development of depression and cognitive dysfunction [49]. Zhang et al. [49] have shown that 4-week exposure to 1-bromopropane reduces the number of BrdU-positive cells in the dentate gyrus and mRNA expression of BDNF and its level in a rat hippocampus. The authors also observed a decrease in the DOPAC/ DA ratio in the striatum after 1-week inhalation of 1-bromopropane. Thus, reduction in DA turnover may attest to a suppression of adult hippocampal neurogenesis under subacute inhalation of this neurotoxic agent.

Acute treatment with MPTP can increase the DOPAC/ DA ratio and the 5-HT levels, while reducing the 5-HIAA/5-HT ratio in the hippocampus of C57BL/6 mice 90 min after injection of neurotoxin [48]. Early studies have shown that neurodegeneration of dopaminergic cells in the substantia nigra occurs several days after an injection of MPTP [50]. Furthermore, He and Nakayama [47] showed that the number of BrdU-positive cells in the subventricular zone and olfactory bulb decreased two days after MPTP treatment, while other authors detected an increase in neurogenesis in the substantia nigra and hippocampus 10, 15 and 21 days after MPTP-induced damage to mice [51, 52]. In the research by Kapitza et al., the locomotor activity of mice returned to normal 7 days after an injection of MPTP [48]. The neurotoxic effects of MPTP are probably accompanied by a regenerative process and changes in the DOPAC/DA ratio. This may attest to a neurogenesis induction similar to the changes that took place after chronic treatment with fluoxetine in our study.

Moreover, the published data regarding the difference in the impact of acute and long-term stress on the DOPAC/DA ratio support our hypothesis on the possible role of DA turnover in neurogenesis in an adult mammalian brain. Robinson et al. [53] observed an increased DOPAC/DA ratio in the medial frontal cortex after a 30-min footshock session with rats. Oneday exposure to social-defeat stress also increased the DOPAC/DA ratio in the medial frontal cortex and nucleus accumbens of mice, but this ratio decreased to its baseline after a 10-day exposure to stress [54]. It should be noted that 21-day prenatal stress decreased the DOPAC/ DA ratio in a rat hippocampus, while a combination of stress and fluoxetine treatment maintained the parameter at its baseline in [55]. We suggest that acute stress or acute injection of a neurotoxic agent stimulates the stem cell pool, which could result in an attenuation of the consequences of these damaging factors. However, long-term exposure to a damaging factor may lead to a decline in neuroprotective mechanisms or drug tolerance. Chronic treatment with fluoxetine or another antidepressant may activate these mechanisms and contribute to the overcoming of the tolerance.

It is important to note that chronic treatment with amitriptyline and fluoxetine resulted in an even further increase in the hypothalamic level of NA compared with UCMS, highlighting the possible contribution of stress reaction to the efficacy of chronically administered antidepressants. Huang and Herbert [56] observed that the circadian rhythm of corticosterone secretion is important for the triggering of a stimulant effect from fluoxetine with respect to neurogenesis in the dentate gyrus of the hippocampal formation in adult rats. Moreover, fluoxetine did not influence the proliferation of neuronal precursors in the hippocampus of adrenalectomized rats compared to intact animals, but everyday administration of corticosterone led to a restoration of the neurogenic effects induced by fluoxetine in adrenalectomized rodents [56]. Like fluoxetine, amitriptyline enhances cellular proliferation in the presence of dexamethasone or cortisol [45]. Thus, this data supports the hypothesis that chronic stress plays an important role in the triggering of the effects of amitriptyline and fluoxetine under long-term treatment.

## CONCLUSIONS

The antidepressant-like effect of single fluoxetine and amitriptyline administration coincided with a decrease in the 5-HT turnover in the hippocampus or a tendency toward similar changes. However, chronic treatment with amitriptyline and fluoxetine increased the hypothalamic levels of NA. A similar effect was also observed after the UCMS procedure, but it proved more pronounced after chronic administration of the antidepressants. The dynamics of 5-HT, NA, and DA neurotransmission changes are in line with the literature data about the role of monoamines in this process. The observed pattern of neurochemical changes after chronic and single administration of amitriptyline and fluoxetine had different characteristics. It would appear that the changes in the DA and 5-HT turnover observed after chronic treatment with the studied antidepressants (opposite to those caused by UCMS) correlate with evidence in the literature regarding neurogenesis in adults. Further research into the mechanisms underlying the interaction between chronic stress, monoaminergic systems, and neurogenesis in the hippocampus may help address the problem of stress-induced therapeutically resistant depression and facilitate the search for new molecular targets for the development of antidepressants.

## Abbreviations

5-HT: serotonin (5-hydroxytryptamine)
5-HIAA: 5-hydroxyindoleacetic acid
HVA: homovanillic acid
DA: dopamine
DOPAC: 3,4-dihydroxyphenelacetic acid
mPFC: medial prefrontal cortex
MPTP: 1-methyl-4-phenyl-1,2,3,6-tetrahydropyridine
NA: noradrenaline
UCMS: unpredictable chronic mild stress
